# Brief cognitive tests validated in Peru for detection of cognitive impairment A systematic mapping of the scientific literature

**DOI:** 10.1590/1980-57642020dn14-020006

**Published:** 2020

**Authors:** Nilton Custodio, Eder Herrera-Pérez, Rosa Montesinos, David Lira, Tatiana Metcalf

**Affiliations:** 1 Servicio de Neurología, Instituto Peruano de Neurociencias, Lima, Perú.; 2 Unidad de Investigación de Deterioro Cognitivo y Prevención de Demencia, Instituto Peruano de Neurociencias, Lima, Perú.; 3 Escuela de Postgrado, Universidad Católica San Pablo, Arequipa, Perú.; 4 Unidad de Investigación Molident, Universidad San Ignacio de Loyola, Lima, Perú.; 5 Servicio de Rehabilitación, Instituto Peruano de Neurociencias. Lima, Perú.

**Keywords:** cognitive impairment, dementia, Alzheimer’s disease, frontotemporal dementia, brief cognitive tests, comprometimento cognitivo, demência, doença de Alzheimer, demência frontotemporal, testes cognitivos breves

## Abstract

Brief cognitive tests (BCTs) are necessary for early detection of cognitive impairment, particularly in primary care settings.

**Objective:**

This report describes a systematic review of BCTs evaluated in Peruvian populations.

**Methods:**

We used systematic mapping techniques to identify articles on screening tests for cognitive impairment involving Peruvian subjects. We included studies published in English and Spanish up to 2018. We reviewed 6 reference databases within the Virtual Health Library network, as well as the Web of Science, Scopus (MEDLINE), and EMBASE databases.

**Results:**

Ten out of 447 articles met the inclusion criteria. Studies included both outpatient (9) and community-based (2) samples. Eligibility criteria of the studies were similar. Although different protocols were applied, the diagnostic criteria were standardized. For discrimination between dementia and controls, IFS (AUC: 0.99) and ACE (AUC: 0.95 to 1.00) showed superior performance, as did the M@T (AUC: 1.00) and CDT-Mv (AUC: 0.94 to 1.00) for discriminating between Alzheimer’s disease (AD) and controls.

**Conclusion:**

The available evidence is limited. However, our analysis of national data suggests that the ACE may be a good choice whenever it can be applied to Peruvian patients. Alternatively, the M@T and IFS can be used for screening patients with suspected AD or FTD, respectively.

## Resumo.

Testes cognitivos breves (TCBs) são necessários para a detecção precoce do comprometimento cognitivo, particularmente nos serviços de atenção primária.

**Objetivo:**

Este artigo descreve uma revisão sistemática dos TCBs avaliados em populações peruanas.

**Métodos:**

Utilizamos técnicas de mapeamento sistemático para identificar artigos sobre testes de triagem para comprometimento cognitivo envolvendo indivíduos peruanos. Incluímos estudos publicados em inglês e espanhol até 2018. Revisamos 6 bancos de dados de referência na rede da Biblioteca Virtual em Saúde e no Web of Science; Scopus (MEDLINE) e banco de dados EMBASE.

**Resultados:**

Dez dos 447 artigos preencheram os critérios de inclusão. Os estudos incluíram amostras ambulatoriais (9) e comunitárias (2). Os critérios de elegibilidade entre os estudos foram semelhantes. Embora os diferentes protocolos tenham sido aplicados, os critérios diagnósticos foram padronizados. Para a discriminação entre demência e controles, INECO Frontal Screening (IFS) (AUC: 0.99), Addenbrooke’s Cognitive Examination (ACE) (AUC: 0.95 to 1.00) mostraram desempnho superior, assim como o Memory Alteration Test (M@T) (AUC: 1.00) o Desenho do relógio (CDT-Mv) (AUC: 0,94 a 1,00) para discriminação entre a doença de Alzheimer (DA) e os controles.

**Conclusão:**

As evidências disponíveis são limitadas. No entanto, nossa análise com dados nacionais sugere que o ACE pode ser uma boa opção sempre que possível com pacientes peruanos. Alternativamente, o M @ T e o IFS podem ser usados ​​para rastrear pacientes com suspeita de DA ou DFT, respectivamente.

Dementia is defined as a marked decline in cognitive function that is severe enough to affect social and occupational functioning.^1^ The most common form of dementia is Alzheimer’s disease, although many other conditions can cause dementia, including vascular dementia, Frontotemporal dementia (FTD), dementia with Lewy bodies, Parkinson’s with dementia, and mixed dementia. Other less common disorders can also cause symptoms of dementias.^2^

At the end of 2010, there were 35.6 million people with dementia globally; 58% of whom were in middle-income countries. Dementia is projected to rapidly increase in the near future, by nearly 63% (relative to 2010 levels; i.e. to 65.7 million cases) in 2030 and by 71% (relative to 2010 levels; i.e. to 115.4 million cases) in 2050.^3^ Dementia is therefore a growing global health concern, especially in low- and middle-income countries (LMIC) where there are major gaps in the availability of services. In Latin America, population ageing further adds to poor socioeconomic conditions in producing a critical scenario.^4^

Early identification of cognitive impairment can allow for timely intervention, thereby slowing down the progression of the disease. This can be especially beneficial in cases where the treatment does not modify the natural course of the disease.^5^ Thus, the need for further research on early diagnosis of Alzheimer’s disease, as well as on identifying the root cause of neurodegeneration and cognitive impairment, has been stressed in various studies as a means of prevention and control of disease progression globally.^6^^,^ 7

A study reported that generalists detect <40% of patients with cognitive impairment, with failure rates proving highest in preclinical and prodromal stages than in patients with established dementia (56% versus 19%).^8^ In this context, access to brief cognitive tests (BCTs) is essential to increase detection in outpatient consultations, whether as a screening test or to confirm a diagnosis in persons suspected of having cognitive impairment.^9^^-^ 11

One of the main drivers behind this study was to inform the medical community of existing validated BCTs for cognitive impairment. Understanding which BCTs have been validated would also help to identify existing research gaps and hence the research needs which might be prioritized in the future.

The study objective was to create a systematic map to identify and describe the properties of BCTs in Peru.

## METHODS

### Study design

We conducted a systematic mapping review of scientific articles on BCTs published in indexed journals based on a protocol that addressed the overarching objective of the study with the following research questions:

*RQ1. What are the BCTs validated in Peruvian populations according to scientific literature in both English and Spanish from inception to 2018?*
*RQ2. What type of research is being conducted on BCTs in relation to screening and diagnosis for cognitive impairment (e.g. methodology, type of disorder, age, sex, education)?*

The properties of BCTs are: 1) ease of use, simplicity and short administration time (no more than 5-10 minutes for primary care doctors and specialists, respectively) and training; 2) objective evaluation, unequivocal (free of complex instructions) and straightforward assessment (without the need for additional instruments); 3) user-friendly and ecologically friendly (does not generate rejection in the individual being evaluated; 4) validated within the context in which it will be applied (independent of personal , sociodemographic, ethnic, or cultural characteristics); and 5) flexible to be adapted for use in other contexts.^12^

For the purpose of this study, the following BCTs were considered: 1) *Addenbrooke’s Cognitive Examination* (ACE);^13^ Eurotest^14^ and its South-American versions as the Peruvian monetary test^15^ or Chilean monetary test;^16^ *Executive Battery 25* (EB25) and its abbreviated version AEB12;^17^ *Free and Cued Selective Reminding Test* (FCSRT);^18^ *Frontal Assessment Battery* (FAB);^19^ *INECO Frontal Screening* (IFS);^20^ *Memory Alteration Test* (M@T);^21^ *Memory Impairment Screen* (MIS);^22^ *Memory, Fluency, and Orientation* (MEFO);^23^ *Mini-Mental State Examination* (MMSE);^24^ *Montreal Cognitive Assessment* (MoCA);^25^ Clock Drawing Test (CDT);^26^ and *Test Your Memory* (TYM).^27^

### Data collection

The following databases were queried: Web of Science (primarily including MEDLINE and SciELO Citation Index), Scopus, MEDLINE, Excerpta Medica dataBASE (EMBASE), and Virtual Health Library (including LILACS, IBECS, CUMECS, BINACIS, PERNAL and MINSA). We used a broad set of search terms as follows: (*root “Peru”*) AND (complete and abbreviated search terms of each of the BCTs considered in this study) OR (“cognitive impairment” OR “cognitive decline” OR “dementia”). The latter terms were used in order to avoid excluding studies that evaluated different BCTs to those identified *a priori*. No temporal limitations were applied.

#### Search process

The following search string was used on each of the reference databases. A total of 883 articles were obtained. Search results were put into an electronic database and duplicate articles eliminated. The remaining articles were reviewed by two independent researchers - a neurologist specialized in cognitive disorders (NC) and an epidemiologist specialized in systematic reviews (EHP), through the screening of title, abstract, and keywords.

At this point, the same two researchers filtered the 17 references, independently screening them for the full content of each paper. A final set of 15 references were produced and included in the study.

The following data were collected: a) study subjects (population, participant eligibility criteria, sample and comparison group); b) methodology (study design type); c) diagnostic procedures (ruling out other causes of cognitive impairment, tests, procedures and diagnostic criteria utilized; and d) BCT performance according to comparison groups (cut-off points, area under the ROC curve, sensitivity, specificity, % correctly classified, probability coefficient, predictive values, Cronbach’s alpha, and Pearson’s coefficient).

The *Pfeffer Functional Activities Questionnaire* (PFAQ) was also included for evaluating functioning and for the screening of dementia.^28^^,^ 29

### Statistical analysis

All studies selected and relevant information extracted was put into a database and further analyzed using STATA version 15. With the goal of comparing BCT performance, we utilized the area under the receiving operating characteristic (ROC) curve (AUC).

### Ethical aspects

The protocol was approved by the ethics committee of the Instituto de Medicina Tropical “Daniel Alcides Carrión” of the Universidad Nacional Mayor de San Marcos under approval number CIEI-2018-020.

## RESULTS

### Study selection

**Figure 1:**
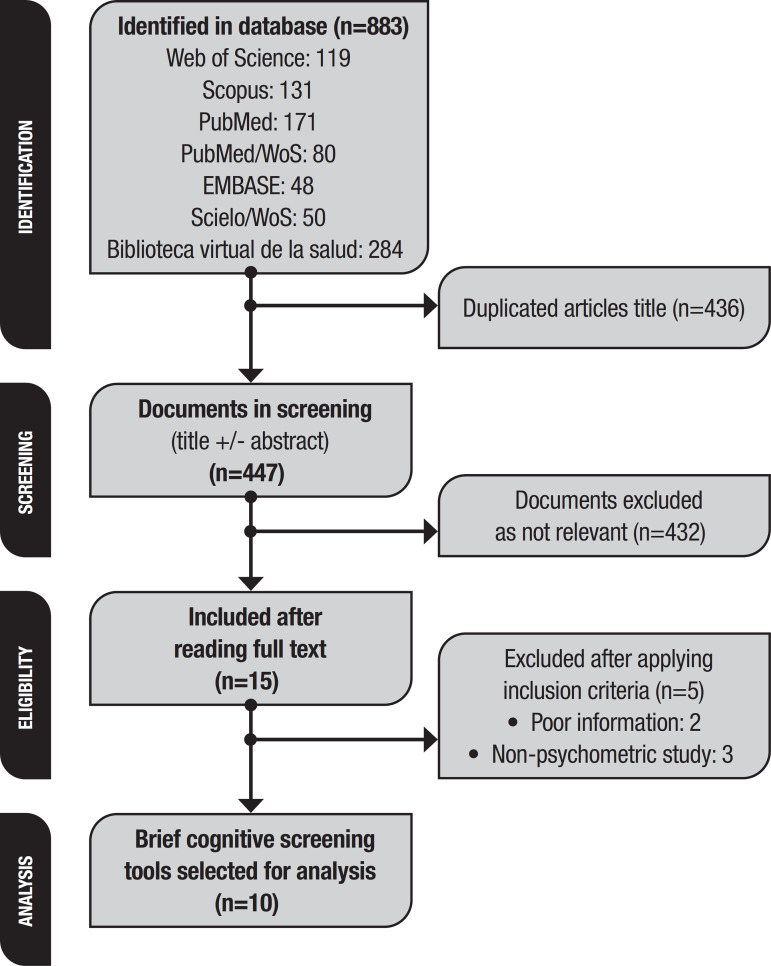
Flowchart of study selection process

The search resulted in the retrieval of 883 articles, of which 436 duplicates were removed. A further 432 were excluded based on screening of the title and abstract. A total of 15 eligible articles were reviewed using the full-text version of the papers. Of these, five were subsequently excluded (two for insufficient information and three that were not focused on evaluating psychometric test performance). A total of 10 studies were thus selected for inclusion in the systematic mapping [Figure 1]. These studies assessed only seven BCTs, namely, the MMSE, ACE, CDT-Mv, FAB, IFS, M@T, and the Peruvian monetary test (PMT).

### Study sample characteristics

Of the total studies included, two were secondary analyses from databases produced for other studies with different objectives. The studies included involved clinical samples from outpatient settings, where all but two were conducted using community-based residential samples^39^ or in elderly day care centers, all of which were located in the capital city of Lima. Differential diagnosis included dementia, dementia of the Alzheimer type (DAT), frontotemporal dementia (FTD), Parkinson’s disease dementia (PPD), mild cognitive impairment (MCI), and depression (Table 1).

**Table 1:** General study characteristics.

Author	Study participants	Study group	Control group	Intergroup homogeneity
Oscanoa T, 2004^48^	Patients	AD (31)	No AD (31)	Yes: Age and education No: Sex**
Custodio N, 2011^49^	Patients	Dementia (103)	Negative controls (107)	Yes: Age and sex No: Education
Custodio N, 2012^50^	Patients Healthy subjects	DAT (40) FTD (18)	Healthy	Yes: Sex and education No: Age
Herrera-Pérez E, 2013^†^ 51	Patients Healthy subjects	Dementia (102): DAT (53), FTD (26), and PDD (23)	Healthy (70) Depression (21)	Yes: Sex and education No: Age
Custodio N, 2014^52^	Patients	DAT (90) MCI (45)	Negative controls (180)*	Yes: Sex and education No: Age
Custodio N, 2015^53^	Patients Healthy subjects	bvFTD (28)	Healthy (20)	Yes: Age, sex and education
Oscanoa T, 2016^15^	Sick Patients Healthy subjects	Dementia (42)	Without dementia (42)	Yes: Age, sex and education
Custodio N, 2016 [A]^54^	Patients Healthy subjects	DAT (35) FTD (34)	Healthy (48)	Yes: Age, sex and education
Custodio N, 2016 [B]^†‡^ 55	General population	Dementia (103)	Negative controls (107)	Not evaluated
Custodio N, 2017^‡^ 56	General population	early DAT (81) aMCI (45)	Negative controls (121)	Yes: Sex and education No: Age

† secondary analysis studies;

‡ community-based sample;

* subjects recruited in non-neurological consultations;

** based on calculations made by authors for the purpose of this study; AD: Alzheimer's disease; MCI: mild cognitive impairment; aMCI: amnestic mild cognitive impairment; DAT: dementia of the Alzheimer type; PDD: Parkinson's disease dementia; FTD: frontotemporal dementia; bvFTD: behavioral variant frontotemporal dementia; negative controls: subjects with negative results after applying diagnostic protocol used to identify study subjects.

We characterized studies in terms of the type of controls: 1) healthy participants; 2) participants who did not have the study disease; and 3) participants that had the study disease ruled-out (negative controls). We evaluated whether the comparison groups within each study were “comparable” in relation to basic criteria of sociodemographic variables (“intergroup homogeneity”). Results revealed that some study groups were not “comparable” in terms of age (four studies), sex (one study), and educational level (one study). However, for three studies, the comparison groups were comparable for all of these sociodemographic variables (Table 1).

Eligibility criteria common to the included studies were: age (particularly >60 years of age), years of education received, the absence of disorders that could affect performance in executing the psychometric tests applied in each of the studies, sensory disorders (vision and hearing) or non-sensory disorders (motor or physical). Only one study included as exclusion criteria use of medications that affect cognitive performance (analgesic opioids, decongestants, anti-spasmodics, anticholinergics, antidepressants, antiarrhythmics, antipsychotics, antiemetics, anxiolytics, and valproate) (Table 2).

**Table 2:** Study eligibility criteria.

		Oscanoa 2004	Custodio 2011	Custodio 2012	Herrera- Pérez 2013	Custodio 2014	Custodio 2015	Oscanoa 2016	Custodio 2016 [A]	Custodio 2016 [B]	Custodio 2017
Age (years)		≥ 60	> 65	> 65	> 55	> 60	≥ 50	≥ 60	> 60	> 60	> 60
Education (years)		≥ 1	≥ 1	≥ 4	≥ 6	≥ 6	≥ 4	NR	≥ 4	≥= 0	≥ 4
Language		NR	Spanish	Spanish	Spanish	Spanish	Spanish	NR	Spanish	Spanish	Spanish
Lack of:	Visual impairment*	✓	✓	✓	✓	✓	✓	✓	✓	✓	✓
	Auditory impairment*	NR	✓	✓	✓	✓	✓	✓	✓	✓	✓
	Motor impairment	hands**	NR	NR	NR	NR	NR	NR	NR	NR	NR
	Physical impairment*	NR	NR	✓	✓	✓	✓	NR	✓	✓	✓

* affects ability to conduct the evaluation;

** impedes writing or reading tasks;

ü: reported. NR: not reported; NS: reported, but not specified.

With regard to the diagnostic protocols applied in each of the studies included, the presence of ruling out potential causes for secondary cognitive impairment was reviewed, revealing the Beck’s Depression Inventory as the main tool used to rule out depression. Similarly, study protocols for carrying out laboratory tests, neuroimaging, and specialized evaluations were reviewed. One of the studies failed to report information on these data tests. Serological testing for syphilis and ELISA-HIV was also included. Likewise, a standardized neuropsychological battery included the *Rey Auditory Verbal Learning Test*, *Logical Memory Subtest* of the revised *Wechsler Memory Scale*, *Trail Making Test* A and B, the *Rey-Osterrieth Complex Figure test,* the *Boston naming test*, *Wisconsin Card Sorting Test*, *Letter-Number* (subtest of the *Wechsler Adult Intelligent Scale III*) and *Digit Span* (Table 3).

**Table 3:** Diagnostic protocol applied in studies selected.

	Oscanoa 2004	Custodio 2011	Custodio 2012	Herrera-Pérez 2013	Custodio 2014	Custodio 2015	Oscanoa 2016	Custodio 2016 [A]	Custodio 2016 [B]	Custodio 2017
**Ruling out causes of secondary cognitive deficits**										
• Depression	GDS ≤11	NR	BDI ≥13	BDI ≥ 13	NR	BDI ≥13	NR	BDI≥ 13	BDI≥ 13	BDI ≥ 13
• Delirium	✓	NR	NR	NR	NR	NR	NR	NR	NR	NR
• Sensory disorder	✓	NR	NR	NR	NR	NR	NR	NR	NR	NR
• Addiction/substance abuse	NR	NR	✓	✓	✓	✓	NR	✓	✓	NR
• Use of medications that impair cognitive performance	NR	NR	NR	NR	NR	NR	NR	NR	NR	✓
• Cerebrovascular pathology	✓	NR	✓	✓	✓	✓	NR	✓	✓	✓
• Subdural hematoma	NR	NR	✓	✓	✓	✓	NR	✓	✓	✓
• Severe TBI	NR	NR	✓	✓	✓	✓	NR	✓	✓	✓
• Neuroinfections*	NR	✓	✓	✓	✓	✓	NR	✓	✓	✓
• Vitamin B12 deficiency	NR	✓	✓	✓	✓	✓	NR	✓	✓	✓
• Hypothyroidism	NR	✓	✓	✓	✓	✓	NR	✓	✓	✓
• Liver disease	NR	NS	✓	✓	✓	✓	NR	✓	✓	✓
• Chronic kidney disease	NR	✓	✓	✓	✓	✓	NR	✓	✓	✓
• Dementia of vascular origin	NR	NA	HIS > 4	HIS > 4	HIS > 4	HIS > 4	NR	HIS > 4	NR	HIS > 4
• Laboratory tests	NR	✓	✓	NS	NR	✓	NR	✓	NS	✓
• Imaging tests	CAT, all	NCCAT, all	CAT and/or MRI, all	CAT and/or MRI, all	NR	CAT and/or MRI, all	NR	CAT and/or MRI, all	CAT and/or MRI, all	CAT and/or MRI, all
Functional capacity assessments	Katz ADL	PFAQ	PFAQ	PFAQ	PFAQ	PFAQ	NR	PFAQ	PFAQ	PFAQ
**Specialized neuropsychological assessment techniques**										
• Medical	Neurologic	NR	Neurologic	Neurologic	Neurologic	Neurologic	Geriatric	Neurologic	Neurologic	Neurologic
• Neuropsychological	✓	✓	✓	✓	✓	✓	✓	✓	✓	✓
• Neuropsychological test battery	NS	NR	✓	✓	NR	✓	NR	✓	✓	✓
• Executive function battery	NR	NR	NR	NR	NR	✓	NR	NR	NR	NR
• Neuropsychiatric assessment battery	NR	NR	NPI	NPI	NR	NPI	NR	NPI	NR	NR

Regarding the diagnostic criteria applied in each of the studies, the DSM-IV and the *National Institute of Neurological and Communicative Disorders and Stroke-Alzheimer's Disease and Related Disorders Association* (NINCDS-ADRDA) were the standards for diagnosing dementia and Alzheimer’s disease. Similarly, we report the criteria utilized in a consistent manner for diagnosing other types of dementia. In this respect, the criteria utilized to classify the severity of the patients were the *Clinical Dementia Rating* (CDR), *Alzheimer’s Disease Assessment Scale-cognitive subscale* (ADAS-cog), and the *Global Deterioration Scale* (GDS) (Table 4).

**Table 4:** Diagnosis and severity criteria applied in studies selected.

	Oscanoa 2004	Custodio 2011	Custodio 2012	Herrera-Pérez 2013	Custodio 2014	Custodio 2015	Oscanoa 2016	Custodio 2016 [A]	Custodio 2016 [B]	Custodio 2017
**Diagnostics**										
• Dementia	DSM-IV	NE	DSM-IV	DSM-IV	NR	DSM-IV	DSM-IV	DSM-IV	DSM-IV	DSM-IV
• Mild cognitive impairment	NA	NA	NA	AAN	Petersen Criteria	NA	NA	NA	NA	Petersen Criteria
• Alzheimer's disease	NINCDS-ADRDA	NINCDS-ADRDA	NINCDS-ADRDA	NINCDS-ADRDA	NINCDS-ADRDA	NA	NA	NINCDS-ADRDA	NINCDS-ADRDA	NINCDS-ADRDA
• Vascular dementia	NA	NINDS-AIREN	NA	NA	NA	NA	NA	NA	NINDS-AIREN	NA
• Frontotemporal dementia	NA	Neary Criteria	Neary Criteria	Neary Criteria	NA	Neary Criteria	NA	Neary Criteria	Neary Criteria	NA
• Lewy body dementia		McKeith Consortium	NA	NA	NA	NA	NA	NA	McKeith Consortium	NA
• Parkinson's disease	NA	NA	NA	UK-PDSBB	NA	NA	NA	NA	NA	NA
**Severity**										
• Criteria	CDR	NR	ADAScog	ADAScog	GDS	CDR	GDS	CDR	CDR	CDR
• Results	CDR 1: 32.3% CDR 2: 38.7% CDR 3: 29.0%	NR	ADAScog mild-moderate	ADAScog mild-moderate	GDS 4-5	CDR 0.5-1	GDS 3-5	NS	CDR 1: 39.8% CDR 2: 34.9% CDR 3: 25.0%	NS

NR: not reported; NS: reported, but not specified; NA: Not applicable; AAN: American Academy of Neurology for diagnosing mild cognitive impairment; ADAScog: Alzheimer's Disease Assessment Scale-cognitive subscale; CDR: Clinical Dementia Rating; Neary Criteria: Clinical consensus criteria for diagnosing frontotemporal dementia; Petersen Criteria: diagnostic criteria for mild cognitive impairment; DSM-IV: Diagnostic and Mental Statistical Manual of Disorders, 4^th^ edition; GDS: Global Deterioration Scale; McKeith Consortium: diagnostic criteria for dementia with Lewy bodies; NINCDS-ADRDA: National Institute of Neurological and Communicative Disorders and Stroke-Alzheimer's Disease and Related Disorders Association; NINDS-AIREN: National Institute of Neurological Disorders and Stroke - Association Internationale pour la Recherche et l'Enseignement en Neurosciences for diagnosing vascular dementia; and UK-PDSBB: United Kingdom Parkinson's Disease Society Brain Bank for diagnosing Parkinson's disease.

### BCT performance analysis

With regards to the BCTs evaluated, we observed that for discriminating between dementia and controls, the IFS (AUC: 0.99) and ACE (ABC: 0.95 to 1.00) showed superior performance to that observed by the FAB (AUC: 0.95), CDT-Mv (AUC: 0.94), and MMSE (AUC: 0.74 to 0.97). For discrimination between dementia of the Alzheimer type and controls, the M@T (AUC: 1.00) and CDT-Mv (AUC: 0.94 to 1.00) showed superior performance to that observed for the MMSE (AUC: 0.72 to 1.00). For discriminating between FTD and controls, the IFS (AUC: 0.98) showed superior performance to that of the FAB (AUC: 0.73) (Table 5).

**Table 5:** Psychometric properties of brief cognitive tests evaluated in studies selected.

		Study group	Reference group	Cut-off point	AUC	Sensitivity (%)	Specificity (%)	Correctly classified (%)	LR+	LR-	PPV (%)	NPV (%)	Cronbach's alpha	Pearson's coefficient
**ACE** ^†^	Custodio, 2012	Dementia (AD+FTD)	Control	86/100	1.000	100	100	NR	NR	NR	NR	NR	0.82	0.85^‡^
	Herrera-Pérez, 2013	Dementia	Depression	85/100	0.997	90.00	100	98.37	NR	NR	NR	NR	NR	NR
		Dementia	Control	90/100	0.948	97.00	67.00	NR	NR	NR	NR	NR	NR	NR
**VLOM/ACE**	Custodio, 2012	FTD	AD+Control	2.84	NR	100	100	NR	NR	NR	NR	NR	NR	NR
		FTD+Control	AD	4.92	NR	100	100	NR	NR	NR	NR	NR	NR	NR
**MMSE**	Custodio, 2011	Dementia	Control	25/30	0.972	100	85.40	92.70	6.87	0.00	NR	NR	NR	NR
	Herrera-Pérez, 2013	Dementia	Depression	27/30	0.887	67	88.00	NR	NR	NR	NR	NR	NR	NR
		Dementia	Control	28/30	0.895	97	67.00	NR	NR	NR	NR	NR	NR	NR
	Custodio, 2014	MCI	Control	28/30	0.8456	83.89	68.89	80.89	NR	NR	NR	NR	NR	NR
		DAT	Control	24/30	1.000	100	96.67	98.89	NR	NR	NR	NR	NR	NR
		DAT	MCI	24/30	1.000	100	96.67	97.78	NR	NR	NR	NR	NR	NR
	Custodio, 2016 [B]	Dementia	Control	≥ 18^‡^/30	0.74	64.10	84.10	NR	4.00	0.40	24.40	96.70	NR	NR
		AD	Control	≥ 18^‡^/30	0.72	60.00	84.10	NR	3.80	0.50	23.20	96.30	NR	NR
	Custodio, 2017	MCI	Control	21/30	0.6536	90.91	13.33	69.88	1.05	0.68	NR	NR	NR	NR
		DAT	Control	21/30	0.8820	90.91	75.31	84.65	3.68	0.12	NR	NR	NR	NR
		DAT	MCI	21/30	0.8278	86.67	75.31	79.37	3.51	2.07	NR	NR	NR	NR
**CDT-Mv**	Oscanoa, 2004	AD	Control	6/10^‡^	NR	83.90	93.50	NR	NR	NR	92.90	85.30	NR	NR
	Custodio, 2011	Dementia	Control	7/10	0.9437	99.03	83.50	91.30	6.00	0.01	83.00	99.00	0.82	0.85^‡^
	Custodio, 2016 [B]	Dementia	Control	7/10	0.94	89.30	98.10	NR	47.79	0.11	79.30	99.10	NR	NR
	Custodio, 2016 [B]	AD	Control	7/10	0.94	90.00	98.10	NR	48.20	0.10	79.04	99.20	NR	NR
	Custodio, 2017	MCI	Control	8/10	0.687	95.04	31.11	77.71	1.38	0.16	NR	NR	NR	NR
		DAT	Control	5/10	1.000	100	87.65	95.05	8.10	0.00	NR	NR	NR	NR
		DAT	MCI	5/10	1.000	100	87.65	92.06	8.10	0.00	NR	NR	NR	NR
**FAB**	Custodio, 2016 [A]	Dementia (AD+bvFTD)	Control	14.5/18	0.95	97.90	81.10	NR	NR	NR	NR	NR	NR	NR
		bvFTD	DAT	13/18	0.73	82.30	48.5	NR	NR	NR	NR	NR	NR	NR
**IFS**	Custodio, 2015	FTD	NR	NR	NR	NR	NR	NR	NR	NR	NR	NR	NR	NR
	Custodio, 2016 [A]	Dementia (AD+bvFTD)	Control	23.5/30	0.99	97.10	97.90	NR	NR	NR	NR	NR	NR	NR
		bvFTD	DAT	17.5/30	0.98	94.12	94.20	NR	NR	NR	NR	NR	NR	NR
**M@T**	Custodio, 2014	MCI	Control	37/50	0.999	98.33	97.78	98.22	NR	NR	NR	NR	NR	NR
		DAT	Control	27/50	1.00	100	98.89	99.63	NR	NR	NR	NR	NR	NR
		DAT	MCI	27/50	1.00	100	98.89	99.26	40.50	0.000	NR	NR	NR	NR
	Custodio, 2017	MCI	Control	35/50	0.996	99.17	91.11	96.99	11.16	0.009	NR	NR	NR	NR
		DAT	Control	29/50	1.000	100	98.77	99.50	81.00	0.00	NR	NR	NR	NR
		DAT	MCI	26/50	0.996	100	97.53	98.41	NR	NR	NR	NR	0.79	0.79^‡^
**PMT**	Oscanoa, 2016	Dementia	Control	24/31	NR	90.50	83.30	NR	NR	NR	NR	NR	NR	NR

ACE: Addenbrooke's cognitive examination; AD: Alzheimer's disease; AUC: area under the ROC curve; bvFTD: behavioral variant frontotemporal dementia; CDT-Mv: clock drawing test, Manos version; DAT: dementia of the Alzheimer type; FAB: frontal assessment battery; FTD: frontotemporal dementia; PMT: Peruvian money test; IFS: INECO frontal screening test; LR+: positive likelihood ratio; LR-: negative likelihood ratio; M@T: memory alteration test; MCI: mild cognitive impairment; MMSE: mini-mental state exam; NPV: negative predictive value; NR: not reported; PPV: positive predictive value; VLOM: verbal-language/orientation-memory ratio;

† Peruvian version of ACE test, an adaptation of original version developed for Mathuranath and colleagues;

‡ In relation to MMSE.

Comparing performance in discriminating between MCI and controls, the M@T (AUC: 0.99) proved better than both the CDT-Mv (AUC: 0.69) and MMSE (AUC: 0.65 to 0.85). For discriminating between MCI and dementia of the Alzheimer type (DAT), the CDT-Mv (AUC: 1.00) and M@T (ABC: 1.00) had better performance than the MMSE (AUC: 0.83 to 1.00). Finally, for discriminating between dementia and depression, the ACE (AUC: 1.00) performed better than the MMSE (AUC: 0.89) (Table 5).

## DISCUSSION

### Implications

This study assessed seven BCTs (the MMSE, ACE, CDT-Mv, FAB, IFS, M@T, and the PMT). According to our results, the IFS, ACE, M@T and CDT-Mv showed acceptable performance levels for the discrimination of various types of cognitive impairment. Likewise, a data comparison reported in a recently published systematic review^30^ observed that the performance of the ACE in Peruvian samples was similar for the discrimination between DAT and controls (AUC: 0.98).

The majority of the BCTs included evaluate global cognitive efficiency (MMSE, ACE, CDT-Mv and PMT), which have a distinct value in LMICs such as Peru given that the prevalence of cognitive impairment of vascular origin and mixed dementia tends to be greater in these countries.^31^ In turn, the remaining tests focus on evaluating executive functions (FAB and IFS), that better discriminate cases of FTD and vascular dementia or on memory function (M@T) that best discriminate typical cases of DAT.^32^

The level of scientific evidence suggests that in Andean countries such as Peru, there is a lack of community-based studies. Of the total number of studies included, only one was conducted in a community setting, including persons with low levels of education. This represents a valuable opportunity to improve future studies in that diagnostic accuracy for BCTs varies in community and clinical settings.^33^

We evaluated for homogeneity with regard to key sociodemographic variables. Six out of 10 studies were heterogeneous for one of these variables, primarily age - which is considered a direct reflection of age and dementia prevalence.^34^ We considered that the heterogeneity observed for other variables does not compromise validity in the studies given that sex is not a determinant of cognitive performance and that education effects can be adjusted for (in the case of CDT-Mv).^35^^-^ 36

It has been recommended that after a cognitive complaint suggesting the presence of dementia, a generalist should conduct a physical exam, request bloodwork and urine testing to rule out reversible causes for cognitive impairment, all in addition to applying a BCT. In countries like Canada^37^ and Chile,^38^ the primary care physician has a major role in diagnosing dementia. In the case of clinical specialists, their initial evaluation includes a neurologic evaluation and applying a BCT.^39^ In this regard, it is fundamentally important to report the evidence available to help support decision-making in selecting a BCT.

The NICE guide, an international reference, considers a specific BCT set for the initial diagnostic evaluation of a patient suspected of having dementia that includes the *10-point Cognitive Screener* (10-CS), *6-item Cognitive Impairment Test* (6CIT), *6-item screener*, *Memory Impairment Screen* (MIS), Mini-Cog, and the *Test Your Memory* (TYM) assessment.^39^ None of these tests have been evaluated in communities of Andean countries, probably due to the fact that they were not designed to be used in settings with zero to low levels of education in different cultural settings, such as is the case with Peru.

Even though there is limited evidence, data suggests that given the broad spectrum of global cognitive tests and their performance observed, the ACE can be a first line option in screening for cognitive impairment, particularly when the optimal time to administer the test is available (around 20 minutes).^40^^,^ 41 Moreover, the M@T can be a good option to confirm suspected cases of AD, whereas the IFS is suited for detecting FTD or vascular disease.^42^

The clinical utility of the BCTs needs to be analyzed in a rigorous manner. It is indeed a valuable tool for opportune identification of cognitive impairment in primary care settings. It is also important to rule out that obtaining normal results after conducting a BCT should not be considered the only input to rule out a diagnosis of dementia.^39^ This is particularly important in individuals with high levels of education and a good cognitive reserve, or even in cases of recurring depression where a complete neuropsychological evaluation and longitudinal follow-up tend to be clues for establishing a diagnostic.

### Limitations

Akin to all systematic investigations, we faced the challenge of a lack of data in the original studies available.^30^ Consequently, we cannot rely on the validity of the results for rural populations or populations whose native language is not Spanish, since original studies did not include these populations. Additionally, the studies selected did not report data about the reliability (inter-observer and test-retest) and practicality (training demand, administration time, implementation costs) of the tests assessed. Finally, another means of improvement relates to the uniformity of the data reported, since none of the studies covered all the information required for this systematic mapping (see tables).

### Strengths

A recently published systematic review addressed the BCT topic for cognitive impairment,^30^^,^ 33 but does not comprise publications conducted in the Spanish language or indexed in databases such as LILACS. Another distinguishing point in our study is the performance mapping of a BCT set in different subtypes of dementia. Another strength is in reference to the inclusion criteria and detailed evaluation of the rates of diagnostic accuracy, an observation made in other publications.^30^^,^ 43

### Conclusions

Our investigation further supports evidence for the IFS, ACE, M@T and CDT-Mv as viable options for discriminating various types of cognitive decline and identified the ACE as potentially the best alternative screening test evaluating global cognitive impairment. However, more reliable studies are needed that incorporate the diversity of the local setting in Andean countries to provide sound recommendations.

### Recommendations

We stress that there is no assessed BCT providing optimal diagnostic performance to discriminate between the different forms of cognitive impairment. Therefore, primary care physicians should focus on the strategies of selection and prioritization of the BCTs based on their clinical suspicions. Considering the available data, in the Peruvian setting we recommend starting with the ACE whenever possible, subsequently opting for the M@T and IFS in cases of suspected AD or FTD, respectively.

This first recommendation is particularly valid for urban contexts in Peru. For environments outside the Peruvian context, its validity depends on the similarity in sociodemographic conditions. Thus, according to previous reports, we anticipate better validity for Andean countries and Mexico.^44^^,^ 45 Additionally, further investigation on this topic is necessary, particularly for adapting, validating and assessing the several BCTs in each region.^46^

Taking into account the Latin-American context, future investigations should collect data on subjects with different levels of education (including low- educated and illiterate individuals), geographical location (including rural zones),^47^ and mother tongue (including indigenous languages),^43^ as well as report all data about the psychometric properties of BCTs (performance, reliability and practicality). Our study could be used as the basis for generating a checklist for research protocols.

In settings with limited resources for the administration and scoring of BCTs, it is recommended to have digital assessment tools in place that can be self-administered.^43^ Unfortunately, none of the studies included had applied BCTs with these characteristics. Given their greater usability potential for population-based screening, it is necessary to evaluate the performance of computerized BCTs, such as the *Computer Assessment of Mild Cognitive Impairment* or the UCSF Brain Health Assessment, as well as those that are not yet computerized may be administered with the use of information technology such as the IFS, ACE, or the M@T.
